# Successful treatment through staged laparoscopic transgastric endoscopic retrograde cholangiopancreatography for postoperative bile leakage: A case report

**DOI:** 10.1097/MD.0000000000030312

**Published:** 2022-09-02

**Authors:** Chi-Young Jeong, Jung Woo Choi, Jae-Ri Kim, Jae Yool Jang, Jin-Kyu Cho

**Affiliations:** a Department of Surgery, Gyeongsang National University Hospital, Jinju, Republic of Korea; b Department of Internal Medicine, Gyeongsang National University Hospital, Jinju, Republic of Korea; c Department of Surgery, Gyeongsang National University Changwon Hospital, Changwon, Republic of Korea.

**Keywords:** bile leakage, endoscopic retrograde biliary drainage, endoscopic retrograde cholangiopancreatography, laparoscopy, transgastric

## Abstract

Therapeutic laparoscopic-assisted transgastric endoscopic retrograde cholangiopancreatography (LA-ERCP) is a safe and effective technique for patient who are unable to receive endoscopic or percutaneous transhepatic treatment. This procedure shows a high overall success rate in managing pancreaticobiliary complications, comparable to that of ERCP.

**Patient concerns:**

A 51-year-old man had abdominal pain for 2 days. The patient showed acute calculous cholecystitis and acute cholangitis with distal common bile duct (CBD) stones. We performed laparoscopic cholecystectomy and removed the distal CBD stones through CBD exploration.

On the fourth day after the surgery, bile leakage was observed through the surgical drain.

**Diagnosis:**

The patient was diagnosed with postoperative bile leakage based on clinical findings.

**Interventions:**

The patient could not receive ERCP or percutaneous transhepatic biliary drainage because he had severe trismus and limb stiffness after suffering from poliomyelitis. So, we performed LA-ERCP, sphincterotomy, and biliary stent insertion. The fully covered self-expanding metal stent was implanted within the percutaneous gastrostomy site around, and 4 weeks later, the stent was removed during re-ERCP.

**Outcomes:**

The patient was discharged without any complications. There were no long-term complications noted during the 12-month follow-up.

**Conclusion/Lessons:**

Staged LA-ERCP represents a practical strategy for managing bile leakage and offers a novel solution for patients for whom transoral and transhepatic approaches are unsuitable. As a result, clinicians must know techniques for gaining access to the biliary system, such as LA-ERCP.

## 1. Introduction

Postoperative bile leakage is a serious complication following numerous hepatobiliary procedures. Despite the gradual progress in surgical techniques and instruments during the last decade, bile leakage is still observed at 2.6% to 7% of patients after laparoscopic common bile duct (CBD) exploration.^[1–3]^

Endoscopic therapy, which includes biliary stenting and/or endoscopic sphincterotomy, is traditionally considered the best treatment for postoperative bile leakage.^[4]^ Endoscopic management of postoperative bile leakage is technically difficult in patients with changed gastrointestinal anatomy or those who are not suited to a transoral approach; thus, these patients are usually treated with percutaneous transhepatic biliary drainage (PTBD) or surgery.

Several techniques for accessing the pancreaticobiliary tract, including Roux-en-Y gastric bypass, have recently been developed for patients with surgically changed gastrointestinal anatomy due to improved surgical and endoscopic techniques. Altered endoscopic methods have been developed and are used in patients with surgically changed gastrointestinal anatomy, including balloon enteroscopy, laparoscopic-assisted transgastric endoscopic retrograde cholangiopancreatography (LA-ERCP), and percutaneous assisted transprosthetic endoscopic therapy.^[5–7]^

Only a few case reports involving the use of LA-ERCP to treat postoperative bile leakage in patients with changed gastrointestinal anatomy have been published.^[8–10]^ Here, we present the case of a 51-year-old man who developed bile leakage following laparoscopic exploration of the CBD. He was successfully treated with a 2-stage transgastric endoscopic retrograde cholangiopancreatography (ERCP) method using LA-ERCP and transprosthetic endoscopic therapy.

## 2. Case presentation

A 51-year-old man visited the emergency room with abdominal pain of 2 days’ duration. He had severe trismus and limb stiffness after suffering from poliomyelitis. He was hemodynamically stable and afebrile on examination. He could open his mouth approximately 10 mm from the inferior to superior incisors. His arms were locked to his chest, and he could not move them due to limb stiffness. On physical examination of the abdomen, he had severe tenderness in the epigastric area with Murphy’s sign.

The laboratory test results were as follows: white blood cell count, 12,889/mm^3^; hemoglobin, 13.0 g/dL; platelet count, 256,000/mm^3^; albumin, 3.3 g/dL; total/direct bilirubin, 2.26/1.62 mg/dL; alanine aminotransferase, 366 U/I; aspartate aminotransferase, 423 U/I; alkaline phosphatase, 372 U/I; gamma-glutamyl transferase 550 U/L; and C-reactive protein, 10.2 mg/L. Abdominal computed tomography revealed gallbladder (GB) dilatation with a GB stone and dilatation of the CBD with a stone. The patient was diagnosed with acute calculous cholecystitis and cholangitis with distal CBD stones based on these findings. He underwent laparoscopic cholecystectomy and CBD exploration.

**Figure 1.**
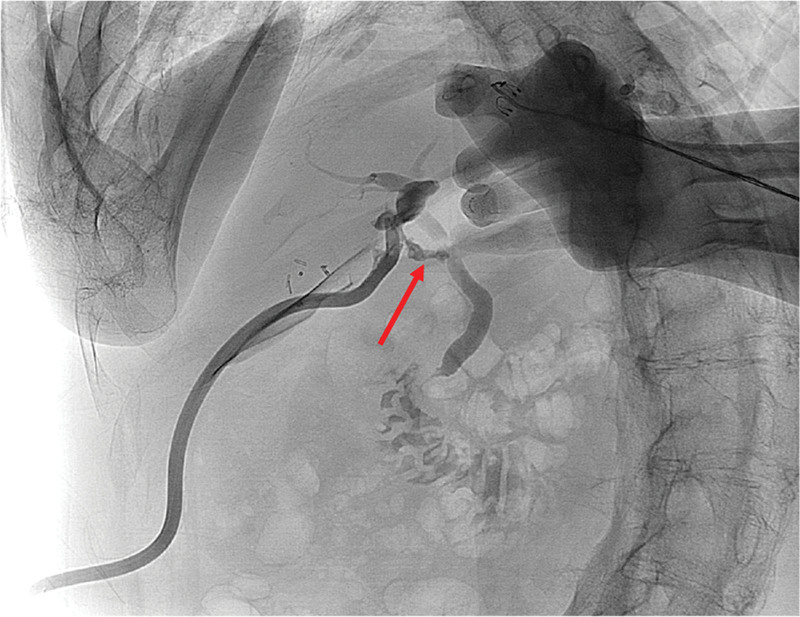
Radiographic view of the surgical drain shows bile leakage at the common bile duct.

On the fourth day after the surgery, bile leakage was observed through the surgical drain (Fig. 1). As a result of his poliomyelitis, he had a limited degree of mouth opening due to trismus and stiffness in both arms across his chest. He could not receive transoral ERCP or PTBD. Therefore, LA-ERCP was performed.

Laparoscopic access was accomplished with the insertion of 4 ports. After the insertion of 3 trocars in the umbilicus and right upper quadrant, a gastrotomy was performed on the anterior side of the mid-body of the stomach, and a purse-string suture anchored the stomach.

**Figure 2.**
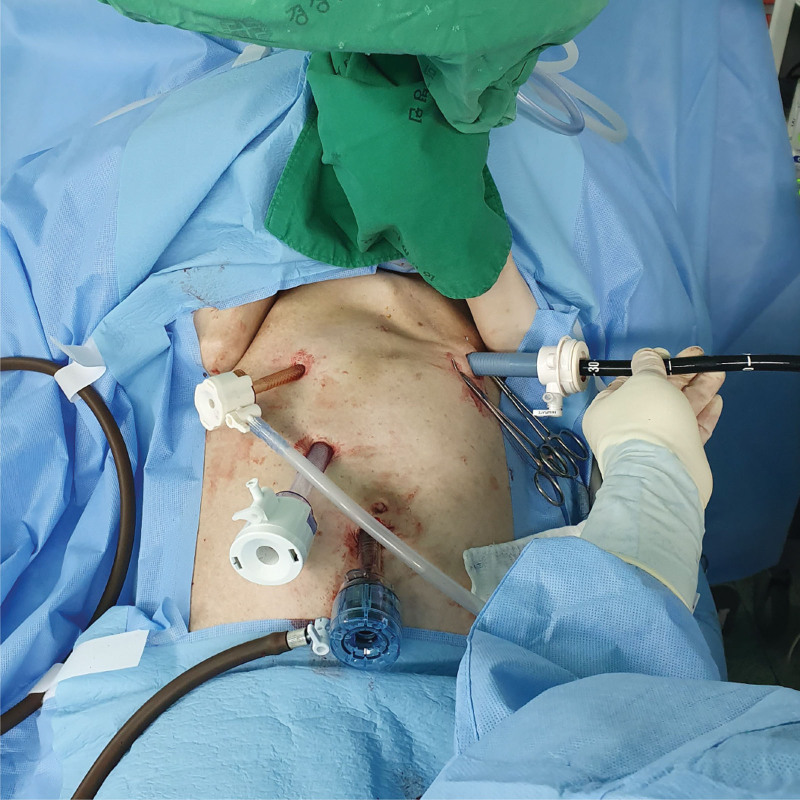
Laparoscopic port position in the operation room. An endoscope is introduced through the 15-mm port, which is inserted in the left upper quadrant.

**Figure 3.**
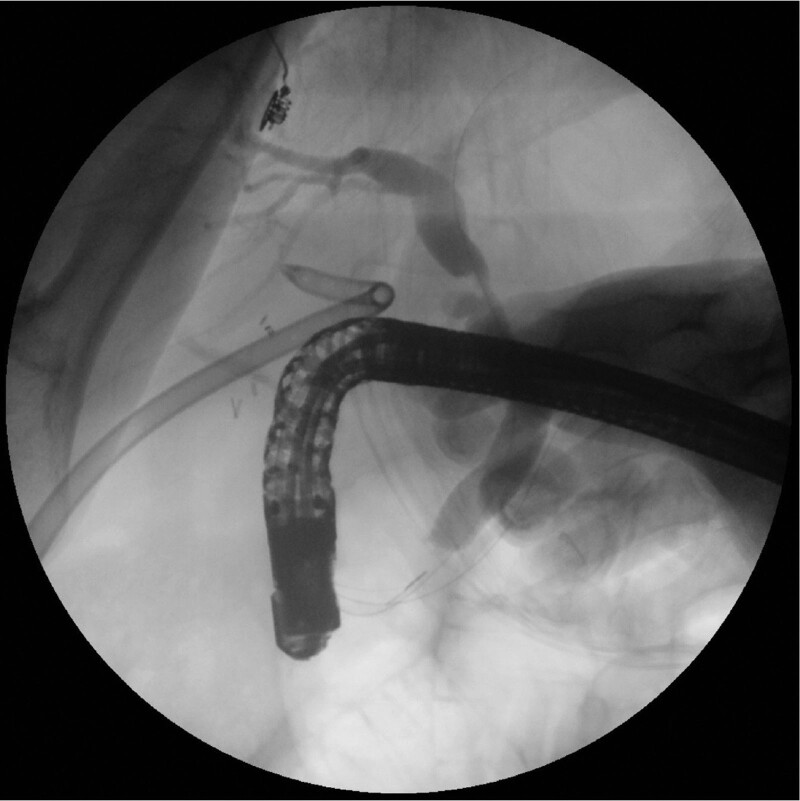
Radiographic view of conventional ERCP performed after antegrade passage of the duodenoscope through the laparoscopic port. ERCP = endoscopic retrograde cholangiopancreatography.

An additional 15-mm port was inserted in the left upper quadrant and gastrotomy was introduced in the midline of the purse-string suture. The stomach was lifted to the anterior abdominal wall after the purse-string suture was tightened. An endoscope was introduced through the 15-mm port (Fig. 2). ERCP, sphincterotomy, and 7-Fr × 10 cm endoscopic retrograde biliary drainage (ERBD) insertion at the leak site were performed (Fig. 3). After ERBD insertion, a 26-Fr balloon bumper percutaneous endoscopic gastrostomy (PEG) catheter was inserted through the 15-mm trocar site for reintervention and stent removal. The bile leakage healed 2 days after the procedure. The patient was discharged without any complications on the fifth day.

**Figure 4.**
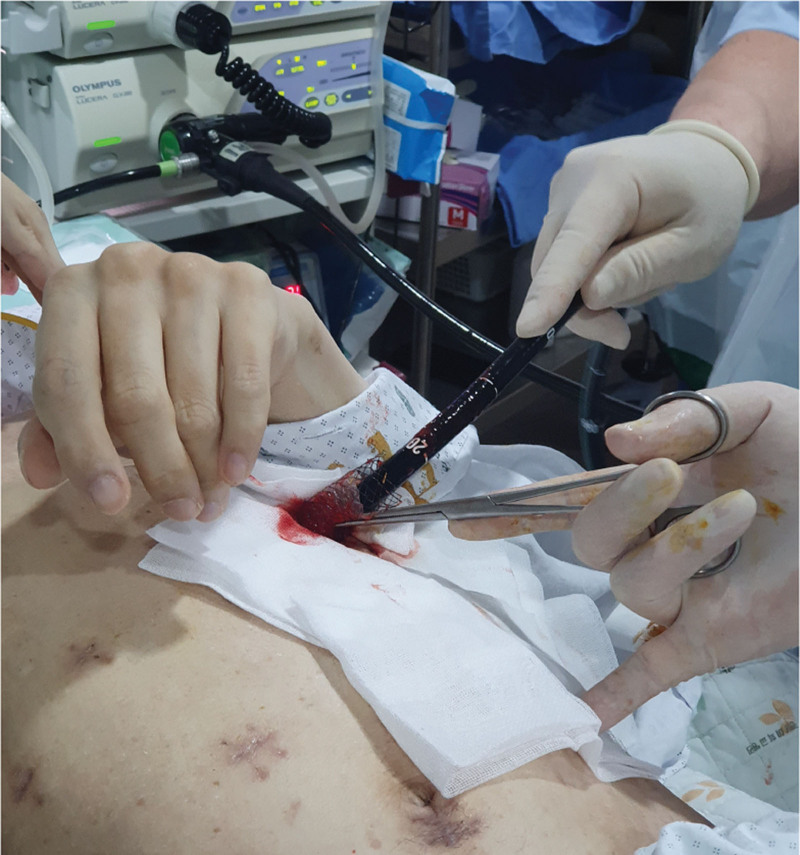
Within the gastrostomy tract, a fully covered self-expanding esophageal metal stent is implanted. An antegrade ERCP is deployed via the stent once it is fully expanded. ERCP = endoscopic retrograde cholangiopancreatography.

About 4 weeks later, the patient was readmitted for ERBD removal. The balloon dilation had been used to increase the size of the percutaneous gastrostomy track. After sequential dilatation of the gastrostomy track, a fully covered self-expanding esophageal metal stent (Taewoong Medical, Seoul, Korea) was deployed within the gastrostomy tract. After the stent was fully expanded, antegrade ERCP was performed through the percutaneous stent (Fig. 4). Under fluoroscopy, bile leakage was not observed. We removed the ERBD. After the ERCP, a 26-Fr balloon bumper PEG catheter was inserted at the gastrostomy site, which was subsequently removed for 2 weeks for tract maturation. The patient was discharged without any complications on the 15th day. There were no long-term complications noted during the 12-month follow-up. This case study was approved by our ethics committee (Gyeongsang National University Hospital, GNUH 2022-04-008) and was performed in accordance with the ethical standards of the Declaration of Helsinki.

## 3. Discussion

This study showed that 2-stage transgastric ERCP is an effective treatment for managing postoperative bile leakage when endoscopic and percutaneous transhepatic approaches are not feasible.

Postoperative bile leakage is a rare but serious complication of severe hepatobiliary surgery, such as liver resection, liver transplantation, cholecystitis, and CBD exploration.

Traditionally, postoperative bile leakages are treated with an endoscopic approach via ERCP and percutaneous transhepatic approaches such as PTBD. ERCP with biliary stent insertion or PTBD is the primary and effective treatment for bile leakage.^[11]^ However, when ERCP and PTBD are not successful or not feasible, treating bile leakage is challenging.

In this case, both endoscopic and percutaneous transhepatic approaches could not be performed due to the patient’s physical disability. We performed LA-ERCP to treat postoperative bile leakage, and the treatment was successful without any complications. Our report suggests that LA-ERCP could be a practical and safe alternative treatment method for patients who cannot undergo endoscopic or percutaneous transhepatic procedures.

The LA-ERCP procedure, which includes a laparoscopy- assisted surgical port insertion into the stomach and percutaneous entry of the endoscope into the duodenum through the port, was initially reported in 2002.^[12]^ In patients with altered anatomies, such as those who have had Roux-en-Y gastric bypass (RYGB), this technique has proven to be an effective alternative treatment. Several studies have demonstrated that LA-ERCP has a technical success rate of over 95% for choledocholithiasis and a minimal complication rate and facilitates the simultaneous administration of endoscopic therapy and cholecystectomy.^[13]^ However, this technique was not widely used in clinical practice until recently, and performance of LA-ERCP was unusual, except in patients with RYGB. This method was most commonly applied to choledocholithiasis treatment in patients who had previously undergone Roux-en-Y gastric bypass for bariatric surgery. Roberts et al^[14]^ first reported LA-ERCP for choledocholithiasis in a patient with a malignant esophageal stricture, which did not allow passage of the endoscope. Here, we reported a second case of LA-ERCP, this time for bile leakage in a patient in whom passage of an endoscope was not possible.

We performed a procedure similar to that reported by Koggel et al,^[15]^ which involves placement of 4 sutures in a diamond-shaped purse string in the gastrotomy site to lift the stomach and prevent gastric contents from spilling into the peritoneal cavity. After termination of LA-ERCP with biliary stent insertion, a 26-Fr balloon bumper PEG catheter was inserted through the 15-mm trocar site for the re-ERCP procedure. Complete closure of bile leakage generally takes 4 to 7 weeks.^[16]^ Therefore, we removed the ERBD stent 4 weeks after the procedure. For passage of the endoscope through the PEG site, sequential dilatation of the PEG site and a fully covered self- expanding esophageal metal stent (Taewoong Medical, Seoul, Korea) were deployed within the PEG tract. After the stent was fully expanded, antegrade ERCP was performed through the percutaneous stent, and the stent was removed successfully.

On the other hand, this procedure does have disadvantages compared to other procedures. First, general anesthesia is required, which may be challenging to perform in patients for whom general anesthesia poses a high risk. Second, procedure- related complications may occur. Most complications are related to the gastrostomy site, but 1 complication, viscus perforation, may occur in about 0.5% of patients and can be fatal, particularly in cases of duodenal perforation.^[17]^ Third, most endoscopists are trained to perform ERCP in the prone position. Therefore, the supine ERCP is more technically challenging. Recently, Osagiede et al^[18]^ reported that performing ERCP in the supine position leads to poorer visualization and cannulation of the CBD.

Although further research is still needed, through this case report, we suggest that staged LA-ERCP may be a feasible alternative treatment if it appears that the PTBD procedure will be difficult or may require several attempts, particularly in patients who cannot receive transoral ERCP.

## 4. Conclusions

In patients with limited access to traditional endoscopic therapy or transhepatic treatment, LA-ERCP can be a practical approach for managing bile leakage after surgery. This procedure could be used as a gateway to natural orifice surgery. Clinicians must identify techniques for obtaining entry to the biliary tree, such as LA-ERCP.

## Author contributions

Conceptualization, Jin Kyu Cho and Jung Woo Choi; data curation, Jae-Ri Kim and Jae Yool Jang; writing—original draft preparation, Jin Kyu Cho; writing—review and editing, Chi-Young Jeong; supervision, Chi-Young Jeong

All authors have read and agreed to the published version of the manuscript.

## Acknowledgments

The authors thank Dr Tae Hyo Kim for his helpful suggestions and critical review regarding the content of the manuscript.
